# Adaptive changes of telocytes in the urinary bladder of patients affected by neurogenic detrusor overactivity

**DOI:** 10.1111/jcmm.13308

**Published:** 2017-08-07

**Authors:** Chiara Traini, Maria‐Simonetta Fausssone‐Pellegrini, Daniele Guasti, Giulio Del Popolo, Jacopo Frizzi, Sergio Serni, Maria‐Giuliana Vannucchi

**Affiliations:** ^1^ Histology and Embryology Research Unit Department of Experimental and Clinical Medicine University of Florence Florence Italy; ^2^ Department of Neuro‐Urology Careggi University Hospital Florence Italy; ^3^ Department of Urology Careggi University Hospital Florence Italy

**Keywords:** bladder lamina propria, detrusor, immunohistochemistry, myofibroblasts, transmission electron microscopy, αSmooth muscle actin, Calreticulin, Caveolin1, CD34, PDGFRα

## Abstract

Urinary bladder activity involves central and autonomic nervous systems and bladder wall. Studies on the pathogenesis of voiding disorders such as the neurogenic detrusor overactivity (NDO) due to suprasacral spinal cord lesions have emphasized the importance of an abnormal handling of the afferent signals from urothelium and lamina propria (LP). In the LP (and detrusor), three types of telocytes (TC) are present and form a 3D‐network. TC are stromal cells able to form the scaffold that contains and organizes the connective components, to serve as guide for tissue (re)‐modelling, to produce trophic and/or regulatory molecules, to share privileged contacts with the immune cells. Specimens of full thickness bladder wall from NDO patients were collected with the aim to investigate possible changes of the three TC types using histology, immunohistochemistry and transmission electron microscopy. The results show that NDO causes several morphological TC changes without cell loss or network interruption. With the exception of those underlying the urothelium, all the TC display signs of activation (increase in Caveolin1 and caveolae, αSMA and thin filaments, Calreticulin and amount of cisternae of the rough endoplasmic reticulum, CD34, euchromatic nuclei and large nucleoli). In all the specimens, a cell infiltrate, mainly consisting in plasma cells located in the vicinity or taking contacts with the TC, is present. In conclusion, our findings show that NDO causes significant changes of all the TC. Notably, these changes can be interpreted as TC adaptability to the pathological condition likely preserving each of their peculiar functions.

## Introduction

The urinary bladder activity is the result of complex interactions involving the central nervous system, the autonomic nervous system and the bladder wall 1. The human bladder wall, similarly to all the hollow organs, is constituted by three main layers: (1) the smooth musculature (named detrusor); (2) the *lamina propria* (LP) incompletely divided into two portions by an irregularly organized and discontinuous *muscularis mucosae*: a deep LP (DLP) or submucosa and an upper LP (ULP) or suburothelium; (3) the surface epithelium (named urothelium).

Although the reservoir and the voiding functions depend on detrusor relaxation/contraction (together with opposite responses by the urethral sphincters), respectively, the studies on voiding disorders have progressively underlined the fundamental contribution of the other two layers to bladder functionality. Two putative mechanisms have been taken into account to explain the appearance of voiding disorders: (1) an augmented efferent activity (the myogenic hypothesis); (2) an abnormal handling of afferent signals (the urothelium‐suburothelium hypothesis) 2. In particular, the urothelium is considered the sensory system that perceives the urine composition and the hydrostatic pressure, and the suburothelium (properly the LP) represents an integrated system able to guarantee the arrival of the correct information to the nerve centres 2, 3.

The LP hosts several types of cells immersed in an extracellular matrix with a complex composition, a rich vascular web and numerous nerve endings 3. The cell distribution and density and the composition of the extracellular matrix have allowed distinguishing two regions in the LP (see above), independently on the presence of a *muscularis mucosae* 3. Immunohistochemical and transmission electron microscopy (TEM) studies of healthy human LP reported the presence of different types of stromal cells whose nomenclature varies among authors. Indeed, these cells were generically called interstitial cells (IC) or, more precisely, telocytes (TC) and myofibroblasts 4, 5, 6. Furthermore, the use of appropriate immunohistochemical labelling and the ultrastructural investigations allowed distinguishing up to three different TC types 4, 6, 7. Several roles have been attributed to the TC. Starting from the common datum that, everywhere, these cells form a 2‐ or 3D‐network, it has been supposed that they constitute the scaffold that keeps together and organizes the connective components (extracellular matrix, cells and blood vessels). Moreover, TC scaffolds might guide the parenchymal cells and/or their precursors to organize in tissues. Further, as these cells show signs of exocytosis and form exosomes, they could be involved in the production of extracellular molecules with trophic and/or regulatory activities. TC also take privileged contacts with the immune cells [for review see refs 8, 9, 10]. Finally, the possibility that TC mediate neuronal responses has been reported 8, 11, 12.

Suprasacral spinal cord injuries of different aetiologies are at the base of neurogenic detrusor overactivity (NDO), a clinical condition characterized by involuntary detrusor contractions during the filling phase causing urinary frequency, urgency and incontinence 2, 13. Although the incontinence is due to anomalous detrusor contractions, increasing evidences localize the mechanisms responsible for the symptomatology in the urothelium and the underlying LP where abnormal afferent signals would be processed 2. The few morpho‐functional studies available show an increase in the LP cell number in rats with spinal cord injury (SCI) 14, an increase in connexin expression 15, 16 and a shift towards a fibroblast phenotype 4 in the cells of the ULP, and an increase in the ULP thickness 17 in the bladder of NDO patients. To note, all these studies have been performed mainly in bioptic specimens at different stages of the disease, and all of them were focused on the ULP 4, 15, 16, 17, 18. Clinically, the possibility to control incontinence is linked to the efficacy of two types of drugs, the antimuscarinic and the botulinum toxin derived molecules. Nevertheless, all the patients develop, sooner or later, resistance to the drug efficacy and are forced to surgery intervention. Although it is still matter of debate the exact functions that TC and myofibroblasts play in regulating the afferent inputs to guarantee an adequate bladder activity, it is a common convincement that changes in these cells have a role in the pathogenesis of altered bladder functionality such that present in NDO.

In the attempt to bring new data to this debate, we collected samples of full thickness bladder wall from a group of NDO patients comparatively homogenous for aetiology, length of the disease and clinical course with the aim to investigate any morphological changes of the three types of TC previously described in the human bladder by using histological, immunohistochemical and ultrastructural methodologies.

## Material and methods

### Subjects and sample collection

A total of eight patients (four females, four males; mean age: 40 ± 2.9 years) underwent to partial/total cystectomy or urinary diversion (Table 1). All the patients had a NDO diagnosis due to the following pathologies: SCI, multiple sclerosis (MS) and myelitis. The choice of the surgery was carried out because the ongoing treatments with botulinum toxin injections into the detrusor and the anti muscarinic drugs orally administered were no longer effective. In addition, the urodynamic parameters, the possibility of urine reflux and personal reasons concurred to the surgery choice. A total of six patients (one females, five males; mean age: 72 ± 3 years) operated for bladder cancer represented the control group for the present work (Table 1). During the surgical procedure, immediately after organ removal, full‐thickness specimens of the bladder lateral wall were obtained from each patient cutting far from the macroscopic lesion and processed for histological, immunohistochemical and TEM studies. All the patients gave written informed consent and the local Ethical Committee approved the study protocol.

**Table 1:** Clinical data

	Sex	Pathology	Site	Age of spinal injury	Age of surgery	Years of drug treatment
Ctrl 1	Female	Cancer	–	–	58	–
Ctrl 2	Male	Cancer	–	–	69	–
Ctrl 3	Male	Cancer	–	–	77	–
Ctrl 4	Male	Cancer	–	–	74	–
Ctrl 5	Male	Cancer	–	–	76	–
Ctrl 6	Male	Cancer	–	–	78	–
Pt 1	Female	SCI	D8	31	44	13
Pt 2	Female	SCI	D5	25	36	11
Pt 3	Male	Myelite	D4	24	41	17
Pt 4	Female	MS	S‐S**	36	52	16
Pt 5	Female	SCI	D8	29	45	16
Pt 6	Male	SCI	D8	29	47	18
Pt 7	Male	SCI	C6	28	35	7
Pt 8*	Male	SCI	D4	18	25	7

Ctrl: control; Pt: patient; SCL: spinal cord injury; MS: multiple sclerosis. *Non‐responder patient to botulinum toxin treatment. **Suprasacral.

### Routine histology and Immunohistochemistry

The full‐thickness specimens were fixed in 4% paraformaldehyde in 0.1 M phosphate‐buffered saline (PBS, pH 7.4) over night (ON) at 4°C, dehydrated in a graded ethanol series, cleared in xylene and embedded in paraffin. The sections were cut (5 μm thick) using a rotary microtome (MR2; Boeckeler Instruments Inc., Tucson, AZ, USA), collected on positively charged slides and processed for either histological and immunohistochemical labelling. The sections were deparaffinized and rehydrated as usual. To evaluate the tissue organization, the sections were stained with haematoxylin–eosin (H&E). For immunohistochemical labelling, the sections were formerly boiled 10 min. in sodium citrate buffer (10 mM, pH 6.0; Bio‐Optica, Milan, Italy) or treated 20 min. at 90–92°C in Tris buffer (10 mM) with EDTA (1 mM, pH 9.0), as appropriate for antigen retrieval. After antigen retrieval phase, the sections were washed in 0.1 M PBS, incubated in 2 mg/ml glycine (AppliChem, Darmstadt, Germany) for 10 min. to quench autofluorescence caused by the elastic fibres and blocked for 20 min. at room temperature (RT) with 1.5% bovine serum albumin (BSA) in 0.1 M PBS. The primary antibodies diluted in 0.1 M PBS were applied ON at 4°C. The day after, the slides were washed in PBS and incubated for 2 hrs at RT in the dark with appropriate fluorochrome‐conjugated (Alexa Fluor 488‐or 568‐conjugated) secondary antibodies diluted 1:333 in 0.1 M PBS. Tissue sections were then thoroughly washed in PBS and mounted in an aqueous medium (Sigma‐Aldrich, St. Louis, MO, USA). For double labelling experiments, after the first incubation as described above, the sections were re‐incubated with a diverse primary antibody and with the appropriate secondary antibody, following the same procedures. To exclude the presence of non‐specific immunofluorescence staining negative controls were performed omitting the primary antibody and, for the M2r antibody, using the relative blocking peptide (AMR‐002‐peptide; Alomone Lab, Jerusalem, Israel). Information on primary and secondary antibody sources and used concentrations is shown in Table 2. The immunoreaction products were observed under an epifluorescence Zeiss Axioskop microscope (Mannheim, Germany) using 488‐ and 568‐nm excitation wavelength for the green and red fluorescent labels, respectively, and the fluorescence images were captured using a Leica DFC310 FX 1.4‐megapixel digital camera, equipped with the Leica software application suite LAS V3.8 (Leica Microsystems, Mannheim, Germany).

**Table 2:** List of the primary and secondary antibodies

	Host	IHC	Producer
Primary antibody			
anti‐PDGFRα	Goat	1:100	Catalogue n. AF‐307‐NA; R&D Systems, Minneapolis, MN, USA
anti‐CD34	Mouse	1:50	Catalogue n. M7165; Dako, Glostrup, Denmark
anti‐αSMA	Mouse	1:500	Catalogue n. A‐2547; Sigma‐Aldrich, St. Louis, MO, USA
anti‐Cav1	Mouse	1:200	Catalogue n. 610406; BD Transduction Labs, Lexington, KY, USA
anti‐Calreticulin	Chicken	1:200	Catalogue n. PA1‐902A; Thermo Scientific, Runcorn, UK
anti‐CD31	Rabbit	1:50	Catalogue n. ab28364; Abcam, Cambridge, UK
anti‐M2r	Rabbit	1:50	Catalogue n. AMR‐002; Alomone Lab, Jerusalem, Israel
Secondary antibody			
anti‐Goat	Donkey	1:333	Invitrogen, San Diego, CA, USA
anti‐Mouse	Goat	1:333	Jackson ImmunoReasearch Laboratories, West Grove, PA, USA
anti‐Chicken	Goat	1:333	Invitrogen, San Diego, CA, USA
anti‐Rabbit	Goat	1:333	Invitrogen, San Diego, CA, USA

### Transmission electron microscopy (TEM)

Specimens comprehensive of the urothelium plus the LP and specimens comprehensive of the LP and the detrusor were fixed in Karnowsky (8% paraformaldehyde in distilled water and 0.2 M PBS containing 0.055 g/l NaPO_4_ and 0.04 M Lysine, added with 0.5% glutaraldehyde) ON at 4°C, post‐fixed with 1% osmium tetroxide in 0.1 M PBS for 2 hrs at 4°C, dehydrated in graded series of acetone and embedded in Epon by using flat moulds. Semi‐thin sections were obtained with a LKB NOVA ultra‐microtome (Stockholm, Sweden), stained with a solution of toluidine blue in 0.1 M borate buffer and observed under a light microscope to check the area of interest and the selected areas were photographed. Then, ultra‐thin sections (50/60 nm thick) of the selected areas were cut using a diamond knife, stained with an alcoholic solution of uranyl acetate in methanol (50:50) per 12 min. at 45°C followed by an aqueous solution of concentrated bismuth subnitrate per 10 min. at RT, examined under a JEOL 1010 electron microscope (Tokyo, Japan) and photographed.

### Quantitative analysis

The CD34 immunoreactive (IR) structures located in the DLP and detrusor were quantified using digitized images (four images/section; two sections/patient) at 40× objective; attention was made to avoid overlapping between adjacent portions. The quantitation was performed in double labelled (CD34/CD31) sections to identify and exclude the endothelial cells that express both markers. Each image was analysed using ImageJ (NIH, Bethesda, ML, USA) to evaluate the intensity of labelling and to quantify the area of the CD34^+^/CD31^−^ structures. Each photograph was converted to a binary image; the threshold value to analyse the structures of interest exclusively was set in images from the control patients and maintained in those of NDO patients. Results were expressed as optical density ± S.E.M.

The total PDGFRα^+^ TC and the PDGFRα^+^/αSMA^+^ TC located in the ULP were counted using digitized images (10 images/section; two not consecutive sections/two specimens/patient) at 100× objective. In NDO patients, the count was made only in the stratified areas. The non‐responder patient was excluded from quantitation. To photograph the entire thickness of the ULP, two consecutive photographs along the vertical axes were taken. Only PDGFRα^+^ cell bodies provided with at least one process were counted in order to exclude other positive cells such as mast cells and immune cells. Results were expressed as total number of PDGFRα^+^ TC, of PDGFRα^+^/αSMA^+^ TC and as a percentage of the latter compared to the first ± S.E.M.

Statistical analysis was performed using Prism 5.0 (GraphPad Software, San Diego, CA, USA) and applying unpaired Student's *t*‐test to compare the two groups. Differences were considered significant when *P* < 0.05.

## Results

In all the patients, the TC located in both LP and detrusor show important immunohistochemical and structural changes compared to controls.

### Upper lamina propria (ULP)

**Figure 1:**
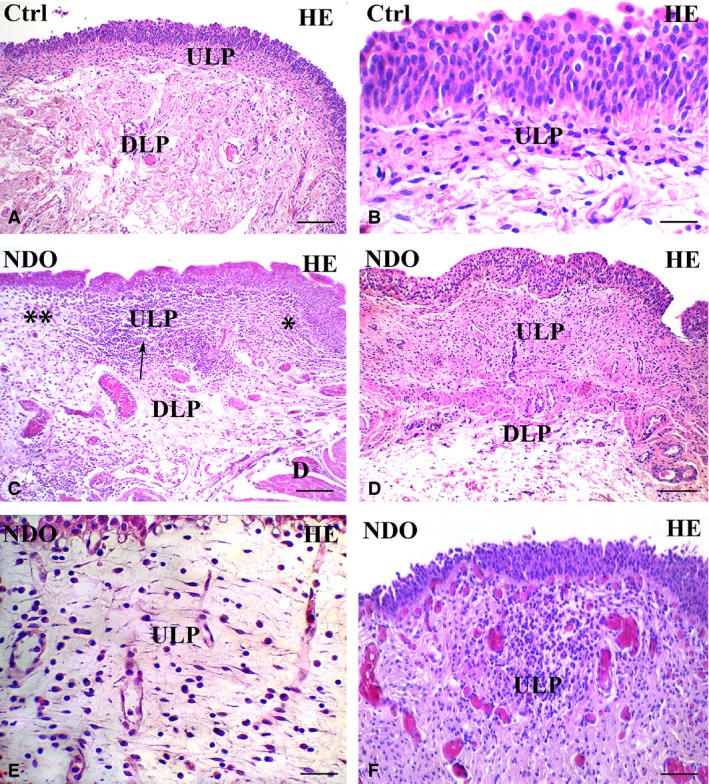
Upper lamina propria (ULP). H&E. (**A, B**) controls. Several rows of cells with an oval body and long, thin processes oriented parallel to the urothelium are stratified and form a network with narrow meshes. (**C**–**F**) NDO patients. The ULP is thicker than in controls: compare **A** with **C** and **D**. In (**C**) alternation of small areas where the cells are stratified and, as in controls, form a network with narrow meshes (asterisk) and areas where the stratification is less evident and the network has large meshes (double asterisks); in between a large group of immune cells (arrow). (**D**) non‐responder patient. The ULP is thicker than in controls and in all other patients (compare with **A** and **C**) and the cellular rows are close to each other leaving a narrow space in between. (**E**) the ULP network has large meshes containing a cell infiltrate and a clear (oedematous) extracellular matrix. The thin TC processes clearly make a network by contacting with each other. (**F**) the cell infiltrate is organized in large groups of immune cells, most of which are plasma cells, surrounded by numerous capillaries. Numerous and hyperemic capillaries are also visible under the urothelium. DLP: deep lamina propria; D: detrusor. Calibration bar: **A, C, D** = 100 μm; **B, E** = 25 μm; **F** = 50 μm.

**Figure 2:**
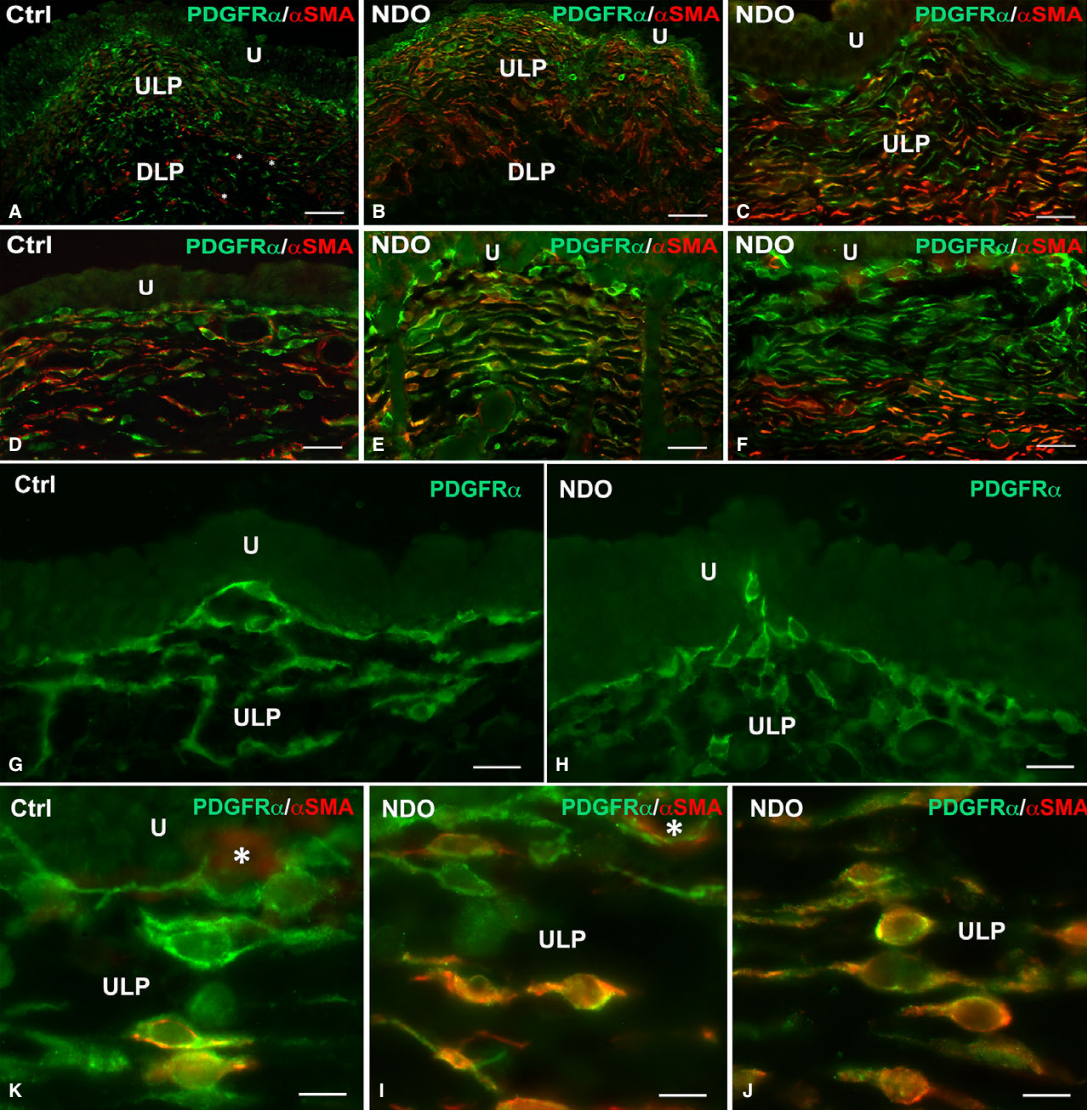
Upper lamina propria (ULP). PDGFRα (green) and αSMA (red) labelling. (**A, D, G, K**) controls. The ULP is made by several rows of cells. Those forming the first row immediately beneath the urothelium are PDGFRα^+^/αSMA^−^ and form a continuous monolayer. The lower rows are made by PDGFRα^+^/αSMA^−^ and PDGFRα^+^/αSMA^+^ cells. Asterisks in **A** indicate three myofibroblasts. (**B, C, E, F, H, I, J**) NDO patients. The suburothelial layer is maintained in all the patients. In the non‐responder patient, the ULP is very thick (**C, F**) and the upper portion is made by several rows of PDGFRα^+^/αSMA^−^ cells while those located in the lower portions are mainly PDGFRα^+^/αSMA^+^ (**C, F, J**). U: urothelium. DLP: deep lamina propria. Calibration bar: **A**–**C** = 100 μm; **D**–**F** = 50 μm; **G, H** = 25 μm; **K**–**J** = 10 μm.

**Figure 3:**
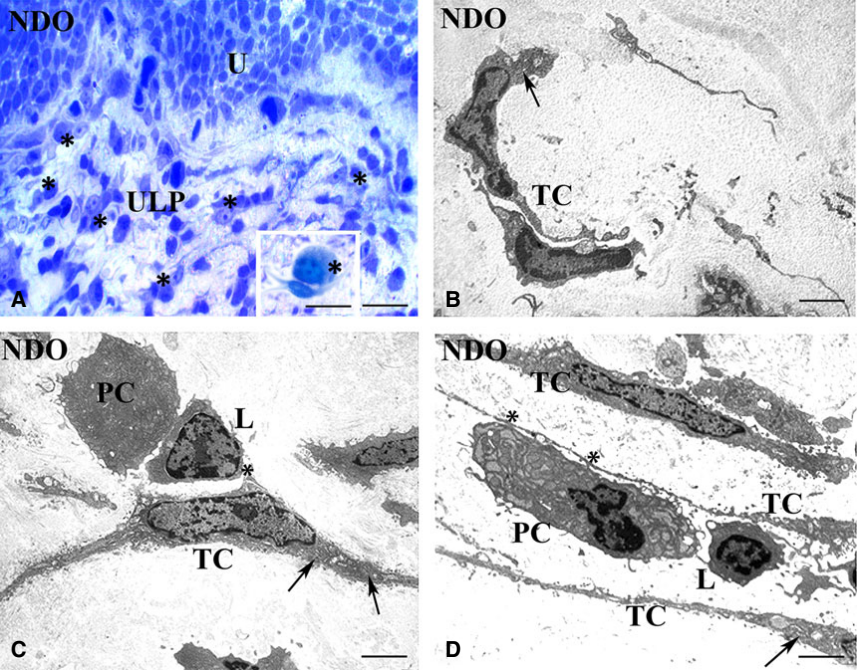
Upper lamina propria (ULP). Typical TC, NDO patients. (**A**) semithin section, toluidine blue stained. An area of the TC network made by regularly stratified cells. Many of these TC have an oval body and long and thin processes. Numerous immune cells (asterisks), many of which are close to the TC, are present. Inset in A: Detail of one plasma cell (asterisk) contacting a TC. (**B**–**D**) transmission electron microscopy. The cells identifiable as typical TC for their small oval body and long and thin processes share numerous RER cisternae in the body and processes (arrows). These TC are often near or make contacts (asterisks) (**C, D**) with the immune cells. U: urothelium; PC: plasma cell; L: lymphocyte; TC: telocyte. Calibration bar: **A** = 25 μm; Inset **A** = 10 μm; **B**–**D** = 1.6 μm.

**Figure 4:**
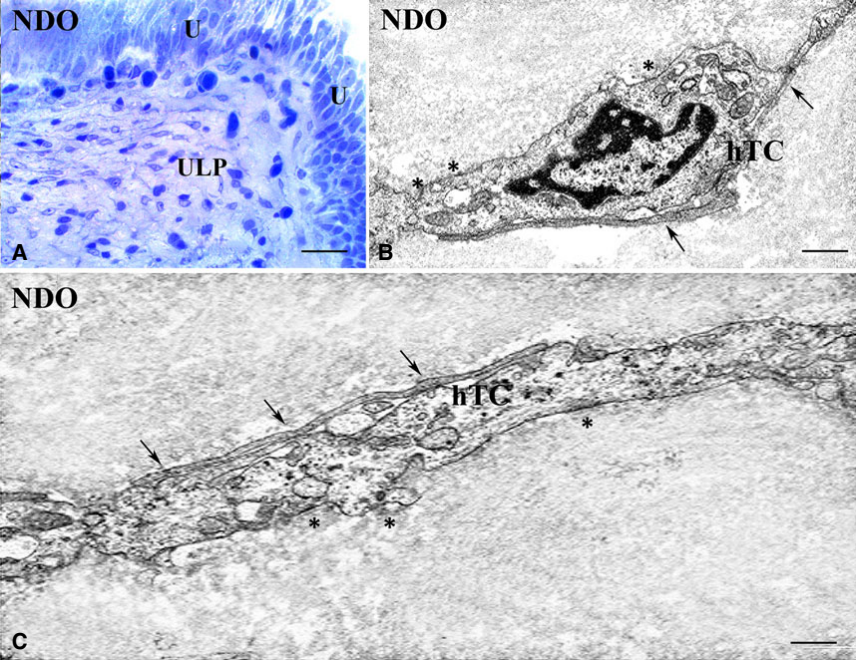
Upper lamina propria (ULP). Hybrid TC, NDO patients. (**A**) semithin section, toluidine blue stained. The TC processes clearly make a network by contacting with each other. Numerous cells, especially those located in the deepest ULP portion and identifiable as hybrid TC, have a large and oval body, a round/oval euchromatic nucleus with a prominent nucleolus, a clear cytoplasm, and thin and long processes. (**B, C**) transmission electron microscopy. The presumptive hybrid TC have an abundant cytoplasm containing sparse, thin filaments, several attachment plaques (asterisks) and many caveolae along the plasma membrane. Arrows indicate the cell‐to‐cell contacts with the processes of other TCs. U: urothelium; hTC: hybrid telocyte. Calibration bar: **A** = 25 μm; **B** = 0.5 μm; **C** = 0.3 μm.

**Figure 5:**
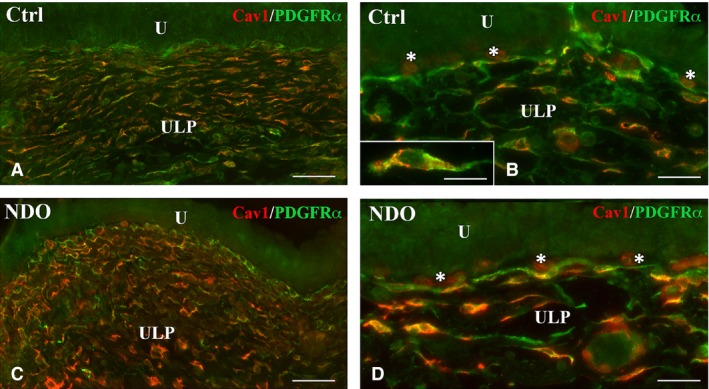
Upper lamina propria (ULP). (**A**–**D and inset in B**) Cav1 (red) and PDGFRα (green) labelling. Compared to controls (**A, B**), the NDO patients (**C, D**) show TC richer in Cav1. Inset (**B**) detail of the Cav1 and PDGFRα distribution in a TC. Asterisks indicate blood capillaries whose endothelium is Cav1^+^ (**B**,** D**). U: urothelium; Calibration bar: **A, C** = 50 μm; **B, D** = 25 μm; Inset **B** = 10 μm.

**Figure 6:**
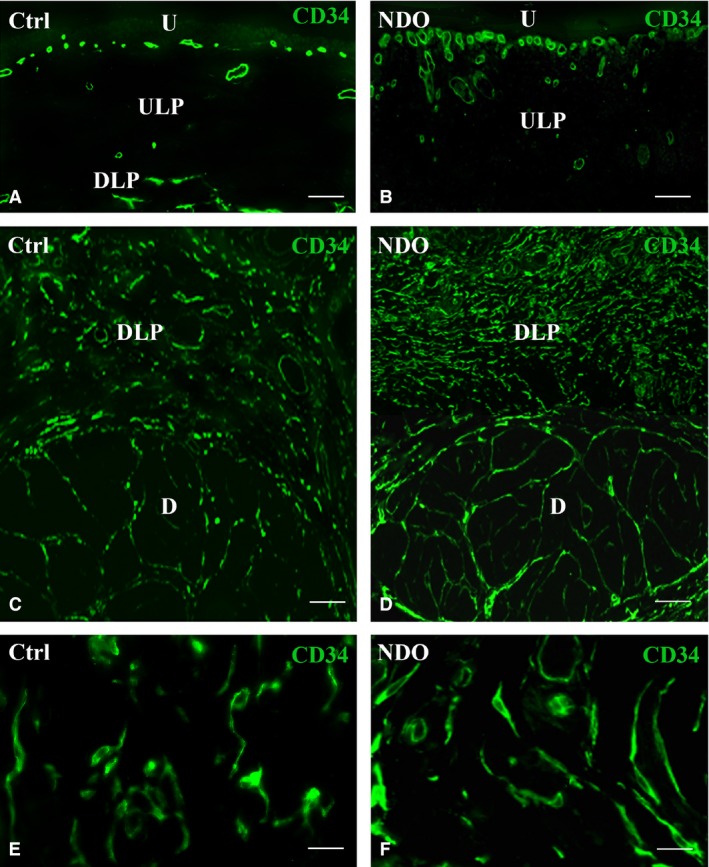
Lamina propria and detrusor. CD34 labelling. (**A, C, E**) controls; (**B, D, F**) NDO patients. In the ULP the endothelial cells only are CD34^+^ (**A**,** B**), while in the DLP and detrusor also the TC are CD34^+^ (**C**–**F**). (**E, F**) details of the DLP‐TC in controls (**E**) and in the NDO patients (**F**). U: urothelium. ULP: upper lamina propria; DLP: deep lamina propria; D: detrusor. Calibration bar: **A**–**D** = 50 μm; **E, F** = 25 μm.

This is the region that shows the most numerous and important modifications (Figs 1, 2, 3, 4, 5, 6). In all the NDO patients, the ULP is thicker than in controls (Figs 1, 2A–F, 5A, C and 6A, B) and, with the exception of the non‐responder patient (Figs 1D and 2C, F), is characterized by alternation of small areas where, similarly to controls (Figs. 1A, B), the cells are stratified and form a network with narrow meshes (Figs 1C and 2B, E) and areas where the stratification is less evident and the network has large meshes (Fig. 1C, E, F). These latter areas are the most represented, and the meshes often contain a cell infiltrate and/or an oedematous extracellular matrix (Figs 1C, E, F, 3A and 4A). The cell infiltrate is either diffuse or organized in large groups of immune cells, most of which are plasma cells (Fig. 3A and inset), surrounded by numerous capillaries (Fig. 1F). As in controls (Fig. 2A, D, G, K), in the ULP of NDO patients two TC types can be identified by immunohistochemistry: the PDGFRα^+^/αSMA‐cells, the presumed typical TC, and the PDGFRα^+^/αSMA^+^ cells, the presumed hybrid TC (Fig. 2B, C, E, F, H, I, J). Underneath the urothelium, the typical TC are only PDGFRα^+^ and, together with blood capillaries (Figs 2K, I and 5B), form a continuous monolayer (Fig. 2A, D, G, K) that is perfectly preserved in all the patients (Figs 2B, C, E, F, H, I and 5C, D). Beneath this monolayer and in continuity with it, a cellular network is present.

In the controls, the ULP network is made by several rows of cells oriented parallel to the urothelium. In each row, the cells make contacts to each other end‐to‐end and, by means of perpendicular processes, with the cells of the under‐ and upper‐lying rows leaving narrow spaces in between (Figs 1A, B and 2G). Moving towards the DLP, the cell rows progressively reduce this parallel organization and the meshes of the net are larger. The two types of TC previously described plus PDGFRα^−^/αSMA^+^ cells identifiable as myofibroblasts make the ULP network (Fig. 2A, D). Both TC types have a spindle‐shaped body, an elongated and heterochromatic nucleus and long and thin processes (Figs 1B and 2G, K). Under the TEM, TC with the typical ultrastructural features alternate with the hybrid TC, that are cells characterized by the presence of small attachment plaques along their plasma membrane 6. Moreover, there are also cells identifiable as myofibroblasts as having a large body rich in cisternae of the rough endoplasmic reticulum (RER), a clear nucleus provided with a prominent nucleolus and long attachment plaques (fibronexuses) on the plasma membrane (data not shown).

In the NDO patients, in those areas apparently intact, the cells forming the network are still organized in stratified rows but a minority of them are only PDGFRα^+^ while most of them also express αSMA (Fig. 2B, C, E, F, I, J). Quantitation of the PDGFRα^+^ TC in the stratified areas shows no difference between controls and NDO patients (9.07 ± 0.77 *versus* 10.97 ± 0.96). On the contrary, the number of the PDGFRa^+^/αSMA^+^ TC (5.93 ± 0.6 *versus* 8.29 ± 0.84) as well as the percentage of these double labelled TC respect to the total TC (0.64 ± 0.03 *versus* 0.76 ± 0.03) show a statistically significant increase in NDO patients. In the infiltrated and oedematous areas, the network is present and still made by the two TC types as in controls. Independently of the area where are located, all the TC have as in controls (Fig. 1B) long and thin processes oriented parallel and perpendicular to the urothelium and contacting to each other, but many of them have a large/oval body and a round/oval euchromatic nucleus provided with a prominent nucleolus (Figs 3A and 4A). Moreover, in the NDO patients, all the TC are richer in Cav1 than in controls (Fig. 5). In the non‐responder patient, the ULP network is particularly thick and forms narrow meshes (Figs 1D, and 2C, F). The upper portion of this network is made by several rows of only PDGFRα^+^/αSMA^−^ TC and the lower portion by many rows of mainly PDGFRα^+^/αSMA^+^ TC (Fig. 2C, F, J).

Under the TEM, many of the typical TC, especially those close to or contacting the immune/plasma cells, contain several RER cisternae in the body and processes and numerous caveolae (Fig. 3B–D). To note, in all the patients, there are no cells closely resembling the hybrid TC described in controls, while, especially in the lower portion of the ULP, where the PDGFRα^+^/αSMA^+^ cells are predominant, there are cells that have a large and oval body, a round/oval euchromatic nucleus with a prominent nucleolus, a clear cytoplasm and thin and long processes by which they contact with each other (Fig. 4B, C). The cytoplasm of these cells contains many sparse thin filaments and along the plasma membrane numerous caveolae and attachment plaques (Fig. 4B, C). Some of these features are similar to those of myofibroblasts, but differently from the latter, these cells are poor in RER cisternae; therefore, we consider them as modified hybrid TC. In favour of this identification, myofibroblasts are still present and show no significant changes either in their αSMA^+^ or in TEM features (data not shown).

In all the patients, as in controls, the CD34^+^ is detected only on the endothelial cells (Fig. 6A, B). Thus, the blood vessels are clearly recognizable and appear hyperaemic, in particular the capillaries immediately located underneath the urothelium (Fig. 6B).

None of the two ULP TC types expresses the M2r.

### Deep lamina propria (DLP) and detrusor

**Figure 7:**
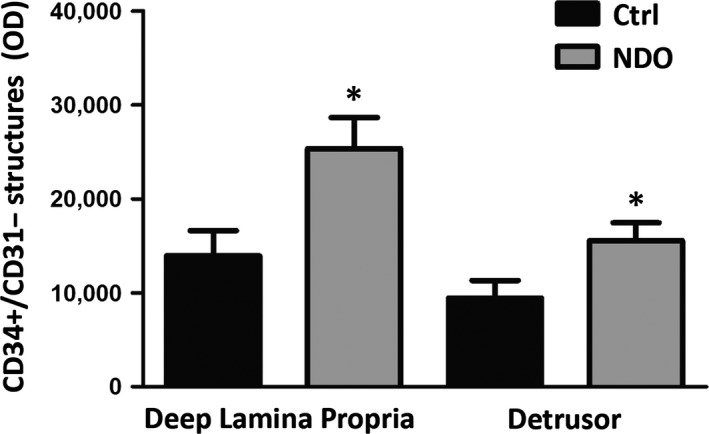
Quantitation of CD34^+^/CD31^−^ in the deep lamina propria and detrusor. The measurements are made in CD34/CD31 double labelled sections and the results concern the only CD34^+^/CD31^−^ labelled structures, that is i.e. the TC. The labelling, expressed as Optical Density is significantly increased in NDO patients: *P < 0.05.

**Figure 8:**
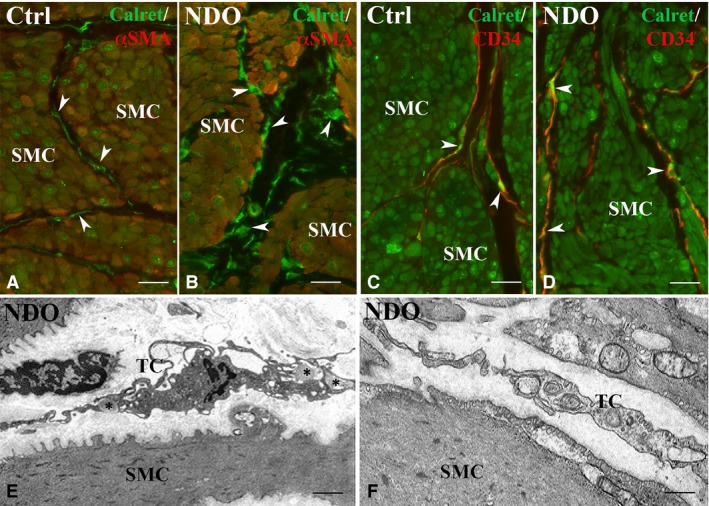
Detrusor. Immunohistochemistry. (**A, B**) Calret (green) and αSMA (red) labelling. In controls (**A**) and NDO patients (**B**) the smooth muscle cells express both markers while the TC (arrowheads) express only Calret. (**C, D**) Calret (green) and CD34 (red) labelling. In controls (C) and NDO patients (**D**), the smooth muscle cells express only Calret while TC express both markers (arrowheads). In NDO patients, the cells identifiable as TC are richer in Calret. Transmission electron microscopy. (**E, F**) NDO patients. The TC are rich in RER cisternae and caveolae. In (**E**), the TC processes embrace bundles of collagen fibrils (asterisks). SMC: smooth muscle cells. Calibration bar: **A**–**D** = 25 μm; **E** = 1.6 μm; **F** = 0.5 μm.

In these regions, there is a single TC population that, both in the patients and controls, is CD34^+^/PDGFRα^−^/αSMA^−^ and forms a network in continuity with that in the ULP (Fig. 6C, D). In all the patients, these TC show a larger cell size (Fig. 6E, F). Quantitation of the CD34^+^/CD31^−^ structures in the DLP and detrusor displays a significant increase of the CD34 labelling in both regions (Fig. 7). Moreover, in the TC located within the detrusor, the Calret labelling is distributed in the entire cell body and processes (Fig. 8A–D). Under the TEM, these TC show an extended RER (Fig. 8E, F) and many caveolae (Fig. 8F). Finally, their processes often embrace bundles of collagen fibrils (Fig. 8E).

None of the DLP and detrusor TC expresses the M2r.

### Urothelium

Both in controls and patients, the urothelium is M2r^+^. The labelling is distributed along the plasmalemma of the urothelial cells with an increasing intensity from the superficial to the deeper cell layers. In the NDO patients, the intensity of the labelling is globally decreased but maintains the same distribution as in controls (Fig. S1A–D).

## Discussion

The present findings demonstrate that the three TC types described in the control human bladder are still present in the NDO patients and have a similar distribution. However, all these TC show morphological changes. The two types located in the ULP, the PDGFRα^+^/αSMA^−^ and the PDGFRα^+^/αSMA^+^ ones, display features of activation. Indeed, under the TEM, the former are enriched in caveolae and RER cisternae and the latter have a euchromatic nucleus carrying a large nucleolus, a clear cytoplasm rich in thin filaments and several caveolae. Both cell types share extended areas of contacts. Moreover, while the TC underlying the urothelium maintains immunohistochemical properties similar to controls, the other TC are richer in Cav1 and, in the stratified areas, the majority of them also express αSMA. The CD34^+^/PDGFRα^−^/αSMA^−^ cells, that are the TC located in the DLP and in the detrusor, also show signs of activation such as an increase of CD34^+^, a large body associated with an intracytoplasmatic Calret positivity and numerous RER cisternae; moreover, the intramuscular TC own many caveolae. All the NDO patients display a cell infiltrate mainly consisting in plasma cells and lymphocytes either sparse or grouped. The plasma cells are constantly located in the vicinity of the TC and often taking close contacts with them. Remarkably, none of the TC modifications indicates degenerative features; rather, these changes suggest a sort of TC adaptation to the pathological environment.

At least two main types of connective tissue cells have been described in the LP of human bladder, the so‐called myofibroblasts 19 and a second population whose identity is still matter of debate. In the attempt to overcome this nomenclature problem, many authors have chosen to call ‘interstitial cells’ (IC) all the stromal cells. In our opinion, this definition is too generic and also improper as the interstitium is the space that separates parenchymal structures while the LP cells reside in a complex matrix made by macromolecules and fibrils and the cells and the matrix, altogether, are responsible for the tissue properties. To solve this problem, we named the stromal cells currently investigated ‘myofibroblasts’ and ‘TC’ on the basis of their ultrastructural features.

NDO is a complex pathology greatly compromising the patient social life. The drugs commonly used to control the overactivity are the antimuscarinic one and the botulin toxins, usually administered in this temporal order. Nevertheless, after a variable period of years (in our case the mean is 13.1 ± 1.5), all the patients become insensitive to these drugs, return incontinent and are forced to surgery intervention. In NDO pathogenesis, a main role is attributed to bladder alteration in handling the afferent signals and the urothelium and the LP would play the bulk of this function. In particular, the urothelium would act as a sensor perceiving chemical and mechanical stimuli generated in the lumen and in releasing several molecules, such as the Ach, in the extracellular spaces and in the underlying LP. In agreement with the literature data 20, 21, we find the M2r not only on smooth muscle cells but also on the urothelium, both in controls and NDO patients (see Fig. S1). Interestingly, in the latter, the expression of this receptor is reduced. This decrease could be due to an excess of ACh production by the urothelium that, in the NDO patients, would develop a condition of hyper‐excitability to local stimuli 13, 22. Still in accordance with the literature, we detect no M2r labelling on the TC and myofibroblasts either in controls or in NDO patients 2.

Noteworthy, in all the NDO patients, the TC forming the monolayer laying the urothelium show features comparable to those of controls. This TC population is quite peculiar either for location or for immunolabelling properties being PDGFRα^+^/αSMA^−^/CD34^−^. Cells with similar immunohistochemical properties have been described immediately beneath the intestinal epithelium 11, 23, and it has been hypothesized that these cells could have a role in controlling the proliferation and differentiation of the overlying epithelium and in maintaining the mucosal homeostasis 23. We believe that in the bladder these TC could play similar functions. In favour of this possibility are: (i) the site of location, (2) the expression of the PDGFRα, the receptor for the platelet derived growth factor that is a major mitogen for many mesenchymal cell types 24, (3) the several findings supporting a role for the TC in regeneration and differentiation of various organs and tissues 25, 26. In summary, the possibility that these TC also play role(s) distinct from those attributed to the other TC types is likely. As mentioned before, and at variance with all the others, in the NDO patients and in our experimental conditions, these TC show no sign of change. We do not have a definitive explanation for this finding. It could be stressed that their location spare these TC from the consequences of the local damages caused by the cell infiltrate. Moreover, if these TC are engaged in cell proliferation and differentiation of the overlying epithelium, the absence of cell death signs in NDO might constitute a further explanation. However, it cannot be excluded the possibility that other changes presently not ascertained occur in these TC.

In the ULP of all the NDO patients, the complex network made up by typical and hybrid TC and myofibroblasts and its continuity with the suburothelial monolayer are conserved. However, because of the presence of the cell infiltrate and oedema, this network is distended and forms large meshes. Intriguingly, most of the immune cells consist of plasma cells. These cells are constantly located in the proximity to the TC and establish contact areas mainly with the typical TC. Cell‐to‐cell contacts and presence of small vesicles (exosomes) in the narrow spaces between TC and immune cells have been described and the possibility that TC have a role in immune‐regulation and immune‐surveillance has been considered 8, 27, 28. In brief, the TC by exocytosis vesicles could release soluble chemo‐attractant molecules, by their long processes act as guides for immune cells, and by their contacts and exosomes production present tissue derived antigens to the immune cells 27. It has been hypothesized that in the NDO, the loss of botulinum toxin efficacy might be due to the development of an immune response consisting in the production of antibodies against the drug and/or its excipients 29. The great number of plasma cells, their constant vicinity to the TC and the high frequency of the cell‐to‐cell contacts between these two cell types found in our patients could be reasonably interpreted as the morphological evidences of the above‐mentioned hypothesis and support a main role of the TC in mediating this immune response. If so, also the signs of cell activation found in the typical TC of the ULP, especially the richness in RER cisternae and caveolae, could be easily explained.

Pointedly, although the TC are engaged in this immune regulation activity and in spite of the presence of an abundant infiltrate, their network seems neither lost nor interrupted in NDO. This 3D scaffold, which extends to the detrusor, guarantees the structural integrity of the bladder wall following the organ distension and relaxation and avoids anomalous wall deformation 6. The maintenance in the patients of this integrity points out the great compliance of the TC in adapting to tissue changes (damages). In particular, the peculiar features of the hybrid TC (*i.e*. the euchromatic nucleus, the large nucleolus, the numerous thin filaments) testify for an enhanced synthetic activity likely aimed to the network preservation. Finally, the presence of the extended cell‐to‐cell contacts we found between hybrid and typical TC and the increase in Cox43 expression reported in NDO 15 further support this TC adaptability.

Functionally, in control conditions, the TC, the myofibroblasts and the numerous nerve endings of the ULP are able to respond to molecules released from the urothelium and behave as an integrated system whose interactions generate afferent stimuli regulating the micturition reflex 2, 3, 30, 31. Likely, the efficiency of this system also depends on the intercellular distances. Our and similar studies 4, 17 have shown a constant increase in the ULP thickness in the NDO patients without a significant increase in cell number. Thus, this increased thickness should cause an augmentation in the intercellular distances, which, in turn, would affect the ULP functional integrity.

The so‐called non‐responder patient represents a case apart. Indeed, in this patient, the marked increase of the ULP thickness is associated with an unusual stratification characterized by numerous rows of only PDGFRa^+^ cells in its upper portion and numerous rows of mainly PDGFRa^+^/aSMA^+^ cells in the deeper portion. Clinically, this patient presents two peculiarities: the youngest age and the refractoriness to botulinum‐toxin treatment. Whether these conditions (and/or others unknown) are responsible for this peculiar TC organization, needs further investigations.

The increase in TC expressing αSMA^+^ present in the stratified areas of all the patients might be interpreted as a shift *versus* a myofibroblast phenotype. Indeed, this possibility has already been reported for TC 4, 17, 32, 33 but the lack of a RER increase and the maintenance of the PDGFRα^+^ exclude similar trans‐differentiations in our specimens.

In the DLP as well as in the detrusor, the TC show signs of activation similar to those described in the TC located in the ULP and maintain the network integrity. Noteworthy, the increase in the cell size associates with an increase in the expression of CD34 and Calret. While the former could be interpreted as the consequence of an increased cell membrane extension, the latter finding is quite intriguing and invites to some speculations on its possible significance. Calret is a complex protein located in the RER, in the nucleus and along the nuclear envelope 34. It acts as chaperon to allow protein folding in the RER but it is also considered a calcium binding protein 35. Keeping in mind some of the roles attributed to the TC, such as the iuxta/paracrine activity, the ability to remodel the collagen fibrils and to control tissue homeostasis 8, it could be speculated that the increased expression of Calret (and, likely, other markers presently not investigated) represents a potentiation of these functions. This possibility is particularly significant considering that this Calret increase happens in the TC located in the detrusor, a compartment of the NDO bladder that shows signs of severe anomalies of both smooth muscle cells and extracellular matrix (personal observations). In this regard, the existence of a functional interaction between TC and smooth muscle cells has been already reported in the uterus 36.

Briefly, the present results show that NDO causes significant changes of all the three TC types present in the human bladder. Importantly, these changes indicate a functional TC activation without cell loss or interruption of the 3D‐stromal scaffold present within the entire bladder wall. Therefore, it can be concluded that all the TC are able to resist to this pathological condition and that their changes are signs of adaptability, likely preserving their peculiar roles.

## Author contributions

CT performed the histological and immunohistochemical experiments, planned the work's steps and analysed the collected data; DG performed the specimens for the TEM. GDP managed the screening of NDO patients, performed the surgery and made available the specimens; JF and SS managed the screening of control patients, performed the surgery and made available the specimens. CT and MSFP prepared the figures; CT, MSFP and MGV wrote the manuscript and made a critical revision of the manuscript; MGV carried out a critical revision of the data obtained, a critical review of manuscript, study concept and design for important intellectual content and obtained the funds. All the authors approved the submitted manuscript.

## Conflicts of interest

The authors declare no conflicts of interest, financial or otherwise.

## Supporting information


**Figure S1** Urothelium and detrusor. M2r labelling. (**A, B**) Urothelium. In controls (**A**), the labelling is distributed along the plasmalemma with an increasing intensity from the superficial to the deeper cell layers. In the NDO patients (**B**), the intensity of the labelling is globally decreased but maintains the same distribution as in controls. (**C**) Urothelium. M2r labelling after pre‐adsorption with the blocking peptide (BP). (**D**) Detrusor. M2r labelling. The M2r labelling is present on the smooth muscle cells. Calibration bar: **A**–**D** = 25 μm.
