# Supplementary figures and images for: Adaptive changes of telocytes in the urinary bladder of patients affected by neurogenic detrusor overactivity
Source: J Cell Mol Med. 2017 Aug 7;22(1):195–206. doi: 10.1111/jcmm.13308 (PMC5742717; doi:10.1111/jcmm.13308)

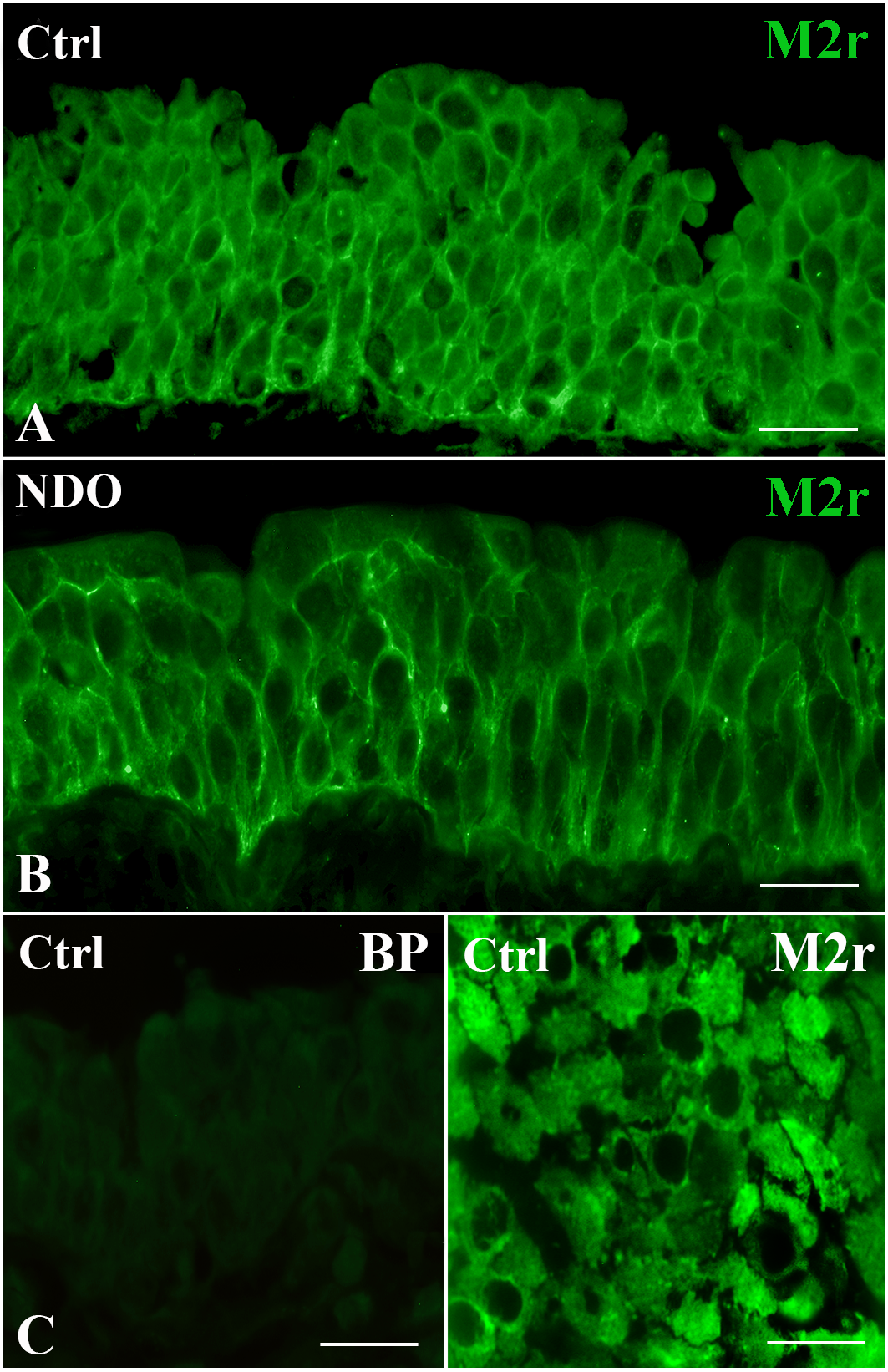

Supplement: Supplementary file 1 — Figure S1 Urothelium and detrusor. M2r labelling. (A, B) Urothelium. In controls (A), the labelling is distributed along the plasmalemma with an increasing intensity from the superficial to the deeper cell layers. In the NDO patients (B), the intensity of the labelling is globally decreased but maintains the same distribution as in controls. (C) Urothelium. M2r labelling after pre‐adsorption with the blocking peptide (BP). (D) Detrusor. M2r labelling. The M2r labelling is present on the smooth muscle cells. Calibration bar: A–D = 25 μm. [file JCMM-22-195-s001.tif]
